# Community Engagement of African Americans in the Era of COVID-19: Considerations, Challenges, Implications, and Recommendations for Public Health

**DOI:** 10.5888/pcd17.200255

**Published:** 2020-08-13

**Authors:** Tabia Henry Akintobi, Theresa Jacobs, Darrell Sabbs, Kisha Holden, Ronald Braithwaite, L. Neicey Johnson, Daniel Dawes, LaShawn Hoffman

**Affiliations:** 1 Prevention Research Center, Morehouse School of Medicine, Atlanta, Georgia; 2 Georgia Primary Care Association, Decatur, Georgia; 3 Georgia Clinical and Translational Science Alliance Community Steering Board, Atlanta, Georgia; 4 Phoebe Putney Memorial Hospital, Albany, Georgia; 5 Satcher Health Leadership Institute, Morehouse School of Medicine, Atlanta, Georgia; 6 Visions, Incorporated, Atlanta, Georgia; 7 Hoffman & Associates, Atlanta, Georgia; 8 Morehouse School of Medicine Prevention Research Center Community Coalition Board, Atlanta, Georgia

## Abstract

African Americans, compared with all other racial/ethnic groups, are more likely to contract coronavirus disease 2019 (COVID-19), be hospitalized for it, and die of the disease. Psychosocial, sociocultural, and environmental vulnerabilities, compounded by preexisting health conditions, exacerbate this health disparity. Interconnected historical, policy, clinical, and community factors explain and underpin community-based participatory research approaches to advance the art and science of community engagement among African Americans in the COVID-19 era. In this commentary, we detail the pandemic response strategies of the Morehouse School of Medicine Prevention Research Center. We discuss the implications of these complex factors and propose recommendations for addressing them that, adopted together, will result in community and data-informed mitigation strategies. These approaches will proactively prepare for the next pandemic and advance community leadership toward health equity.

**Summary**

**What is already known on this topic?**

African Americans are more likely to contract coronavirus disease 2019 (COVID-19), be hospitalized for it, and die of the disease when compared with other racial/ethnic groups. Psychosocial, sociocultural, and environmental vulnerabilities, compounded by preexisting health conditions, exacerbate this health disparity.

**What is added by this report?**

This report adds to an understanding of the interconnected historical, policy, clinical, and community factors associated with pandemic risk, which underpin community-based participatory research approaches to advance the art and science of community engagement among African Americans in the COVID-19 era.

**What are the implications for public health practice?**

When considered together, the factors detailed in this commentary create opportunities for new approaches to intentionally engage socially vulnerable African Americans. The proposed response strategies will proactively prepare public health leaders for the next pandemic and advance community leadership toward health equity.

## Introduction

Racial/ethnic minority populations have historically borne a disproportionate burden of illness, hospitalization, and death during public health emergencies, including the 2009 H1N1 influenza pandemic and the Zika virus epidemic (1–4). This disproportionate burden is due to a higher level of social vulnerability — “individual and community characteristics that affect capacities to anticipate, confront, repair, and recover from the effects of a disaster” — among racial/ethnic minority populations than among non-Hispanic White populations (5). These characteristics include, but are not limited to, low socioeconomic status and power, predisposing racial/ethnic minority populations in general and African Americans in particular to less-than-optimal living conditions. Some racial/ethnic minority populations are more likely than non-Hispanic White populations to live in densely populated areas, overcrowded housing, and/or multigenerational homes; lack adequate plumbing and access to clean water; and/or have jobs that do not offer paid leave or the opportunity to work from home (6,7). These factors contribute to a person’s ability to comply with the mitigation mandates of the coronavirus disease 2019 (COVID-19) pandemic established to reduce risk for infection, such as physical distancing and sheltering in place (8).

The COVID-19 pandemic presents new challenges for public health evaluators, policy makers, and practitioners, yet it mirrors historical trends in health disparities and poor health outcomes among African Americans. African Americans are more likely to contract, be hospitalized, and die of COVID-19–related complications (9–12). Social vulnerability is often compounded by preexisting health conditions, exacerbated during times of crisis (13–17).

Public health leaders are now at a critical juncture to advance health equity among vulnerable African Americans. To advance this health equity, we must first have a comprehensive understanding of the factors that create health disparities and the factors that can contribute to an effective, multilevel response. With this understanding, we can then deploy effective mitigation strategies based on a community-based participatory research framework that fosters and sustains community leadership in the assessment and implementation of culturally appropriate and evidence-based interventions that enhance translation of research findings for community and policy change (18,19). The objective of this commentary is to 1) detail the interconnected historical, policy, clinical, community, and research challenges and considerations central to comprehensively advancing the art and science of community engagement among African Americans in the COVID-19 era; 2) describe The Morehouse School of Medicine Prevention Research Center (MSM PRC) pandemic response strategies, driven by community-based participatory research (CBPR); and 3) discuss community-centered implications and next steps for public health action.

## Challenges and Considerations

### Historical context

Racial/ethnic health disparities have always existed in the United States. Differential health outcomes between African Americans and non-Hispanic White Americans have been part of the American landscape for more than 400 years (20). Many measures of health status have been used to assess differences among racial/ethnic groups; more recently, health researchers have advanced concepts and constructs of health equity and social determinants of health (21). Reaching back to the mid-20th century, the US government documented that African Americans were far more likely than non-Hispanic White Americans to have a wide range of potentially fatal illnesses, including noncommunicable diseases such as type 2 diabetes, asthma, end-stage renal disease, and cardiovascular disease (21). In 1985, the US Department of Health and Human Services published the landmark *Report of the Secretary’s Task Force on Black and Minority Health*, better known as the Heckler report (21). The report documented an annual excess 60,000 deaths among African American and other racial/ethnic minority populations. These underlying determinants can only result in disproportionately adverse health outcomes for racial/ethnic minority populations during the COVID-19 pandemic.

The COVID-19 pandemic is intensified by the long-standing income inequality between non-Hispanic White people and racial/ethnic minority populations. Economists use the Gini coefficient to measure income inequality. Values for this measure range from 0 to 1, with higher values representing greater income inequality. From 1990 to 2018, the Gini coefficient in the United States rose from 0.43 to 0.49 — an increase in income inequality. When income disparities exist along with other disparities (eg, health insurance, employment, education, social justice, access to quality health care), public health pandemics marginalize racial/ethnic minority groups, and this marginalization requires a strong and strategic response (22).

### Policy landscape

Racial/ethnic minority populations are disproportionately affected by COVID-19 (23), as they are by many diseases. In the United States, African Americans, Hispanics/Latinos, Native Americans, Native Hawaiians, and Pacific Islanders are more likely than other racial/ethnic groups to die of COVID-19 (24). The pandemic has not affected all populations equally for several reasons, including social, behavioral, and environmental determinants of health. In addition, economic and social policies have not benefitted all populations equally. Obesity, asthma, depression, diabetes, heart disease, cancer, HIV/AIDS, and many other disorders that put vulnerable populations at greater risk of dying of COVID-19 can often be linked to a policy determinant (25). Air pollution; climate change; toxic waste sites; unclean water; lack of fresh fruits and vegetables; unsafe, unsecure, and unstable housing; poor-quality education; inaccessible transportation; lack of parks and other recreational areas; and other factors play a large role in overall health and well-being (26). These factors increase a person’s stress and limit opportunities for optimal health (27). Too often, public health researchers and practitioners stop at the social determinants of inequities. These social determinants do, indeed, play an outsized role in these human-made inequities, but underlying each one is a policy determinant that should be addressed to improve health equity.

Consider, for example, the problem of asthma among many racial/ethnic minority populations. One community, in East Harlem, one of Manhattan’s poorest neighborhoods, found that a bus depot caused the high rates of asthma among children who lived near it (28). Six of 7 bus depots in Manhattan are located in East Harlem, and East Harlem has the highest rate of asthma hospitalizations in the country (29–31). In another community, the exhaust and dust from the vehicles traveling a major highway that cut through the middle of the community was found to contribute to the high rates of asthma among residents who lived near it (32). In both of these examples, an underlying policy determined the placement of the bus depots and the highway, which led to the eventual health inequities.

Examples of how legislative and policy change can immediately affect the social determinants of health are demonstrated in government and public responses during the first 3 months of the COVID-19 pandemic in the United States. Federal, state, and local policies were implemented to stimulate local economies and infuse communities with free food and direct revenue, including increases in SNAP (Supplemental Nutrition Assistance Program) benefits and expanded unemployment benefits. These initiatives have helped communities and individuals during the crisis. Despite these programs, however, some marginalized African American communities have not benefitted. As the nation adjusts to the “new normal,” it is imperative that the social, economic, and health gaps in these communities also conform to a “new normal” that is driven by new or expanded *and* sustained policies.

### Clinical mechanisms, chronic conditions, and increased risk of COVID-19

African Americans are twice as likely as non-Hispanic White Americans to die of heart disease and 50% more likely to have hypertension and/or diabetes (33,34). This elevated risk increases the likelihood of other complications and death from COVID-19 (35,36). Let us consider, for example, people living with diabetes. Their immune system is depressed overall, because their blood glucose is not well controlled (hyperglycemia) (37). It is hypothesized that hyperglycemia causes an increase in the number of a particular receptor in the lungs, pancreas, liver, and kidneys; this increase impairs the function of white blood cells, which are designed to fight off infections (37). This impairment predisposes the person living with diabetes to an increased risk of bacterial and viral infections. When severe acute respiratory syndrome coronavirus 2 (SARS-CoV-2) enters the lungs by way of this particular receptor, it overwhelms the alveoli (air sacs) in the lungs and disables the exchange of oxygen and carbon dioxide (38). As a result, some people with diabetes may need supplemental oxygen, intubation, and/or admission to an intensive care unit (37). Hyperglycemia in combination with a disease such as COVID-19 makes recovery difficult (37). People with diabetes who are in good mental health, know the names and dosages of their medications, and know their blood pressure, blood glucose, and other laboratory values, such as hemoglobin A_1c_, tend to have better control of their disease and have lower levels of illness and death (16,37). Emphasizing the importance of good blood glucose control to prevent diabetes complications and associated COVID-19 risk is more important now than ever (36–38). Mental health plays a major role in a person’s ability to maintain good physical health and optimally manage their chronic conditions, and mental illnesses may affect the ability to participate in health-promoting behaviors (39).

### Mental and behavioral health

The constellation of stressors triggered by the COVID-19 pandemic undermines the nation’s mental health (40–42). Various disruptions in daily life, coupled with the threat of contracting the deadly virus, is leading some people to experience anxiety and depression, sometimes to the extreme. Reports of family violence and use of suicide prevention hotlines have increased (43,44). Physical distancing, shelter-in-place orders, business and school closures, and widespread unemployment have radically changed ways of life and contributed to a sense of hopelessness, isolation, loneliness, helplessness, and loss (45,46). Pandemic-related factors, including quarantine, have led to posttraumatic stress disorder, confusion, and anger (47). One study indicated that a constant consumption of media reports had detrimental psychological effects on some people (48). If interrelated mental, behavioral, and emotional issues are not adequately addressed, disorders among racial/ethnic minority populations and other vulnerable populations (eg, the medically underserved, homeless, and disabled; inmates in the criminal justice system) will surge and exacerbate disparities (49).

Interrelated COVID-19–related stressors include childcare and safety, elder care, food insecurity, and interpersonal relationships (50). These stressors may trigger aspects of unresolved trauma. Poor coping mechanisms (eg, use of illicit drugs, excessive alcohol consumption, overeating, inadequate sleep) may develop or worsen. In addition to facing chronic stressors, communities of racial/ethnic minority populations often deal with the stigma associated with seeking mental and behavioral health care. A Surgeon General’s report, *Mental Health: Culture, Race, and Ethnicity*, concluded that racial/ethnic minority populations, compared with the non-Hispanic White population, have less access to mental health care, are less likely to receive treatment, and when treated, often receive poorer quality of care (51). As a result, racial/ethnic minority populations often have a greater burden of behavioral disorder–related disability (51). Addressing the multifaceted mental and behavioral health needs of racial/ethnic minority populations in the United States is a complex issue that warrants attention from clinicians, researchers, scientists, public health professionals, and policy makers. It is imperative to recognize the significant role of community leaders in exploring solutions to COVID-19–related mental and behavioral health problems among racial/ethnic minority communities. Their lived experiences are central to the co-creation of pandemic response strategies for these populations.

### Perspectives of community leaders

The realities of research, evaluation, and clinically focused community engagement after the COVID-19 pandemic may change for the foreseeable future. Efforts to initiate and sustain culturally competent engagement of racial/ethnic minority groups previously relied on face-to-face interactions in homes, churches, and other community settings. Social or physical distancing has nearly stopped communities and their collaborators from real-time gathering. These changes challenge the human need for connection and in-person exchange. Although the adjustment has been difficult, the pandemic has resulted in new modes of engagement. Webinar and digital technology are now accessible for most people at low or no cost. Many community residents have newfound capacities to use technology for social and professional interactions as part of daily life.

Current health communication and messaging require community-informed improvements. The use of terms like *sheltering in place, social distancing*, and *flattening the curve* do not naturally resonate with many people. For some, these terms foster anxiety and distrust of systems perceived to separate communities rather than promote COVID-19 mitigation strategies. Community leaders, as well as business and faith leaders, have found themselves in a space of terminology and descriptions that are understood mostly by public health practitioners. Therefore, health literacy and the interpretation of current health conditions are vital.

The pandemic has intensified the economic strains among low-income and moderate-income people and families (52). Low-wage workers, many on the frontlines of the pandemic since it began, have had little to no increase in income (53). African American families who struggled to make ends meet before COVID-19 are now facing dire economic circumstances in making the best decisions for their families. Stressors include, but are not limited to, deciding how to pay rent or a mortgage, paying for food, assisting children with virtual learning, and protecting themselves with minimal or no health care benefits. The mental and behavioral health implications of these problems, along with the economic and practical challenges, have made a fragile ecosystem even more unstable. Low-wage workers in hospitality, food service, and retail industries cannot work from home. Workers who depend on employer-provided health insurance now have the additional burden of how to maintain health insurance coverage (54). Ultimately, lack of adequate access to health care, along with the complex realities of the COVID-19 pandemic, will increase health disparities for socially vulnerable African American employees and their families.

### Local examples of COVID-19 response strategies driven by community-based participatory research

The MSM PRC relies on a deeply rooted, community partnership model that responds to the health priorities of vulnerable African American residents before, during, and after public health emergencies such as the COVID-19 pandemic. For more than 20 years, the MSM PRC has applied dynamic CBPR approaches that focus on prevention, establish partnerships between communities and research entities, and are culturally tailored (6,55–57).

The MSM PRC capitalizes on community wisdom through a community coalition board (CCB) that has governed the center since its inception. The CCB is composed of 3 types of members: neighborhood residents (always in the majority), academic institutions, and social service providers (58). Neighborhood residents hold the preponderance of power, and all leadership seats and are at the forefront of all implemented approaches. Neighborhood resident members are intentionally recruited from census tracts with a high incidence and prevalence of chronic and infectious diseases. The communities served by the MSM PRC are majority (87%) African American, have an average household income of $23,616, and rank lowest among other local communities in other socioeconomic conditions and community neighborhood health factors (55).

The MSM PRC has strategically partnered with the CCB and the community to facilitate health research and related interventions based on a comprehensive understanding of historical, political, clinical, and community considerations. The community governance model was developed to address CBPR challenges that exist when academics are not guided by neighborhood leaders in understanding a community’s ecology, when community members do not lead discussions about their health priorities, and when academics and neighborhood leaders do not work together as a single body with established rules to guide roles and operations (59,60).

The MSM PRC conducts a recurring (every 4 years) community health needs and assets assessment (CHNA^2^) process through the CCB, empowering community members to take on roles as citizen scientists who develop locally relevant research questions and identify priority health strategies (60). The recently completed CHNA^2^ (February 2018) was co-led by neighborhood residents to advance a community health agenda. Survey development, data analyses, and response strategies are reviewed, monitored, and evaluated by the CCB and its Data Monitoring and Evaluation Committee (55). This 7-member committee, established in 2011, is designed to extend the CBPR engagement of CCB members in the work of the MSM PRC. It exists through academic–community co-leadership (a CCB neighborhood resident member and the MSM PRC assistant director of evaluation) of a group of CCB members tasked with leading assessments. For CHNA^2^, members met bimonthly (every other month, when the CCB did not meet) to discuss and inform evaluation and data collection activities and prepare for reporting of evaluation findings and interim results to the broader CCB to determine corresponding respond strategies. CHNA^2^ primary data included surveys administered to 607 community residents. The most frequently cited community health concerns were diabetes, nutrition, high blood pressure, overweight/obesity, and mental health. County-level, top-ranking causes of illness and death, including cardiovascular disease, diabetes, and mental health disorders, align with these community perspectives (61).

CHNA^2^ is relevant, despite being administered before the outbreak of COVID-19. The chronic conditions and health problems identified are those exacerbated by COVID-19 (diabetes, cardiovascular disease, and mental health), thereby making their focus even more relevant to the community.

The mental and behavioral health components of CHNA^2^ were amplified to address the stress and anxiety caused by the pandemic. First, during National Mental Health Awareness Month (May 2020), the MSM PRC convened a virtual forum, *Our Mental and Behavioral Health Matter*s. It was strategically designed to address the culturally bound mental health stigma in racial/ethnic minority communities that is due, in part, to the schism between religion and therapy. The forum also addressed challenges related to social isolation. Concerns centered on how to navigate a virtual mental health checkup and support for parents seeking to help their children process the realities of the pandemic and minimize childhood trauma. Featuring psychologists, researchers, and community- and faith-based pioneers, the forum engaged more than 230 local and national participants. Second, a CCB member representing Fulton County’s Department of Behavioral Health and Developmental Disabilities helped the MSM PRC to develop and disseminate an infographic on mental and behavioral health services for insured and uninsured residents. Third, the MSM PRC will offer annual Mental Health First Aid (62) trainings to community residents and professionals over the next 4 years.

The MSM PRC leads the Georgia Clinical and Translational Science Alliance’s Community Engagement Program, which is designed to advance community-engaged clinical and translational research (63,64). The Program is led by a community steering board adapted from the CCB model and includes co-leaders (faculty and staff, including a community health worker) from Emory University, the Georgia Institute of Technology, and the University of Georgia. The program conducted a webinar, *Community Engagement in the Era of COVID — Opportunities, Challenges and Lessons Being Learned*, in May 2020. The webinar addressed the challenges and opportunities associated with initiating or sustaining community-engaged research during physical-distancing and shelter-in-place mandates. Clinicians, scientists, and community leaders from Atlanta, Athens, and Albany, Georgia, discussed uniquely nuanced issues for urban and rural community engagement and the basic need for social connectedness through virtual navigation of community engagement strategies (eg, via Zoom) and newly expanded access to telehealth medical visits (65). The webinar emphasized the importance of being a credible source of COVID-19 information and linkage across social and economic services, given heightened community anxiety and preexisting mistrust of medical research.

The MSM PRC is a central collaborator in a national initiative led by the National Center for Primary Care at Morehouse School of Medicine and the Satcher Health Leadership Institute, also at Morehouse School of Medicine. The National COVID-19 Resiliency Network is designed to mitigate COVID-19 in racial/ethnic minority, rural, and socially vulnerable communities. The initiative will work with community organizations to deliver education and information on resources to help fight the pandemic. The information network will strengthen efforts to link communities to COVID-19 testing, health care services, and social services through the institution’s leadership in policy, community engagement, and primary care. The MSM PRC’s CCB model will be scaled to collaborate with community organizations in highly affected geographic areas to assess and inventory community assets for COVID-19 testing, vaccination, and other health care and social services through a national community coalition board. The MSM PRC CHNA^2^ model will also be scaled to inform mitigation approaches implemented by community-based organizations through establishment of a centralized inventory of culturally appropriate COVID-19 response strategies, by geography and population vulnerability. Approaches will engage community health workers, who are mission-critical stakeholders, nationally galvanized, and locally deployed.

These MSM PRC activities are founded on long-standing, community-partnered, and informed relationships in response to preexisting health priorities that are simply heightened by the COVID-19 pandemic. Ideally, this CBPR framework is established before a public health crisis. This framework and the practice of identifying community needs and mobilizing strengths are now poised, adapted, and scaled up in response to the COVID-19 pandemic. The continued evolution of the pandemic means that these approaches and solutions must be flexible in response to changing needs and new data.

## Implications for Public Health

Public health practitioners, evaluators, policy makers, researchers, and clinicians with a community-engaged mindset have long understood, grappled with, and proclaimed the complexities of health disparities in the context of historic and current social determinants (66). When considered together, the challenges and realities detailed in this commentary create opportunities for new approaches to intentionally engage socially vulnerable African Americans. The response strategies proposed below reflect the complex web of historical and current policy and clinical, mental and behavioral, and community factors. Use of a CBPR framework undergirds all response strategies proposed.

**Promote local community leadership to proactively inform mitigation strategies.** The importance of CBPR and related needs assessments and response strategies are heightened during the COVID-19 era. Health promotion for chronic conditions such as diabetes, obesity, and cardiovascular diseases may have previously been structured to result in poor health or premature death for racial/ethnic minority populations through reduced or nonexistent access to health care; these conditions now require more immediate attention because they increase vulnerabilities and risks that can lead to poor health outcomes or death. Community knowledge, perceptions, and approaches to culturally responsive mitigation strategies must be prioritized. Carefully constructed local community governance boards that include multidisciplinary leadership (clinical, policy and social service, and research, among others), should be formed to lead assessments toward community and data-informed COVID-19 mitigation strategies for vulnerable populations in highly affected geographic areas.

**Strategically engage public health and community-attuned policy leaders and prioritize community stimulus strategies.** The political landscape calls for public health leadership by mitigation response teams (25). These teams are key informants from the beginning of public health initiatives designed to mitigate the pandemic, and their engagement is essential. They will provide another lens through which to examine the structures and processes that enable inequities to systematically develop and flourish or be eradicated through community co-created responses.

The essential areas of policy for optimal community health are in prioritized economic development, food security, and access to health care protection for vulnerable African American communities. Collectively, these areas present opportunities for intervention in response to chronic disease self-management (clinical), economic strains (community), and health care protections (policy) associated with the COVID-19 vulnerabilities of many African American communities. These essential policy areas represent a proposed foundation that rests on 4 “Es” hypothesized to narrow disparity gaps and offer opportunities for self-sufficiency and community resiliency.

- **E**mploy trained/certified, compensated community health workers, coaches, and ambassadors who are charged with cultural messaging and education, contact tracing, and surveillance toward increased adherence to policies on physical distancing and sheltering in place.
- **E**xpand SNAP programs with vouchers to include the purchase of household and personal care items rather than encouraging recipients to barter for basic care products.
- **E**nhance school lunch programs so that all children receive high-quality, balanced meals throughout the year, regardless of the ability to pay.
- **E**nsure universal broadband internet access to reduce education, health care, and information barriers.

**Cultivate community-informed public health disaster health literacy.** Health literacy concepts, modes, and education must be reframed. The media have newly exposed the lay public to the realities of unequal treatment and unequal pandemic risk. The public is, thereby, witnessing the more rapid connection between who they are, where they live, and who is more likely to suffer from and die of COVID-19. Marketing frameworks for community-based prevention can be used to position community leaders to inform and lead health communication strategies. These marketing frameworks will ensure that messages resonate, engage, and foster action with objectivity and community/cultural sensitivity.

**Foster culturally tailored behavioral and mental health dialogue and response**. Multidimensional prevention education strategies that encourage resilience (positive adaptation to adversity) must be promoted in African American communities. This promotion should involve advocating for proactive self-care, reducing stigma, and encouraging integrated health care. These strategies should be promoted and proactively integrated as cross-cutting components of *any* research and health initiative.

**Prioritize patient-centered medical homes and neighborhood models.** Patient-centered medical home infrastructures that include models of integrated care (mental and behavioral health care services in primary health care settings) can help overcome barriers to comprehensive health care and overall wellness. This model engages comprehensive resources to care for a patient, regardless of race/ethnicity, sex/gender, sexual orientation, language, socioeconomic status, or health insurance coverage. Primary care providers are encouraged to incorporate this model into their practices to decrease illness and death among African Americans at heightened risk of COVID-19 (67,68).

**Redefine essential workers.** Although the accomplishments of first responders — physicians, nurses, scientists, and other people fighting to preserve life — are laudable and undeniable, many African American nonclinical frontline workers, such as maintenance, janitorial, or food processing workers, are excluded from the definition of essential workers. The social vulnerability of nonclinical frontline workers, who often have chronic health conditions that place them at particular risk for contracting COVID-19, should be acknowledged and considered in planning. 

Community and public health leaders in health care, behavioral health, and policy must consider the implications of health inequities among racial/ethnic minority populations, seriously tackle their root causes, and develop culturally responsive COVID-19 strategies for socially vulnerable African Americans. CBPR-driven approaches that elevate marginalized communities as senior partners in planning, implementing, and evaluating strategies will promote community leadership and increase adherence to health communication messages as the COVID-19 pandemic evolves. Efforts should be characterized by strong data (research or evaluation), contextually relevant community engagement strategies, and action (policy, systems, and environmental change approaches). The COVID-19 pandemic has presented an optimal opportunity to reprioritize and sustain approaches toward advancing community engagement of vulnerable African Americans. These new approaches will prepare us for the next pandemic. More importantly, they will foster CBPR leadership in advancing health equity.
